# *LINC00152* acts as a competing endogenous RNA of *HMGA1* to promote the growth of gastric cancer cells

**DOI:** 10.1002/jcla.24192

**Published:** 2022-01-11

**Authors:** Jiayi Chen, Qingfang Zheng, Fang Liu, Han Jin, Xiaoyue Wu, Yang Xi

**Affiliations:** ^1^ Department of Experimental Pathology Ningbo Clinical Pathology Diagnosis Center Ningbo China; ^2^ Institute of Biochemistry and Molecular Biology, School of Medicine Ningbo University Ningbo China; ^3^ Ningbo Haishu District Center for Disease Control and Prevention Ningbo China

**Keywords:** competing endogenous RNA, gastric cancer, *HMGA1*, *LINC00152*, proliferation

## Abstract

**Background:**

Long noncoding RNAs (lncRNAs) play important roles in almost every stage of cancer development. Given the competing endogenous RNA (ceRNA) hypothesis for the regulation of gene expression, we investigated the role of *LINC00152* as a ceRNA in gastric cancer (GC) cells.

**Methods:**

Gastric cancer cell lines were used in this study. Mimics of miRNAs and siRNA were used to evaluate the interaction between *LINC00152* and *HMGA1*. The quantitative real‐time polymerase chain reaction was performed for analyzing gene expression at the transcriptional level. Flow cytometry assay of cell cycle and western blot analysis of related protein expression levels were performed. Online databases such as TCGA and TIMER were used to determine the possibility of *HMGA1* and *LINC00152* as GC markers and their role in immune infiltration.

**Results:**

Treating GC cell lines with *LINC00152* siRNAs downregulated the expression of *HMGA1*. The cell cycle was arrested in the S phase following a reduction in *LINC00152* or *HMGA1* expression, whereas the expression of the cell cycle inhibitor *P27* increased. In this study, we showed that acting as a ceRNA of *HMGA1*, *LINC00152* has the same function as *HMGA1*, considering that it could control the cell cycle and promote GC cell proliferation. The TCGA database showed that *LINC00152* might be used as a diagnostic marker for GC.

**Conclusions:**

These findings provide mechanistic insights into the role of *LINC00152* as a ceRNA to regulate *HMGA1* expression in GC cells, where it can promote the proliferation of the GC cells by regulating the expression of the *P27*.

## Graphical abstract

Given the hypothesis of ceRNA in the gene expression regulation, we investigated the role of LINC00152 as a ceRNA of HMGA1. LINC00152 and HMGA1 formed the ceRNA network with miR15a and Let 7a, and reciprocally regulated. Down‐regulate the expression of LINC00152 and HMGA1 can arrest the cell cycle by regulated the expression of the P27, and inhabit the proliferation in GC cell.

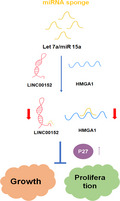

## 1 INTRODUCTION

Gastric cancer (GC) is the most common gastrointestinal cancer; although the incidence rate has declined in recent years, GC is still the second leading cause of cancer, especially in China.^1^ Globally, in recent decades, its incidence rate has been the fourth highest, and its mortality rate is the third highest among cancers in males. Additionally, the incidence rate in males is twice than that in females.^2^ Early‐stage GCs are difficult to detect because the clinical features are not evident, and patients present with nonspecific symptoms.^3^ Currently, about 90% of GC is diagnosed at an advanced stage, and the five‐year survival rate after surgery is below 30%.^4^ A study by Sumiyama found that the five‐year survival rate of early‐stage GC after treatment is greater than 90%.^5^ This implies that the outcome of GC is closely related to the time of diagnosis. Therefore, determining the specific mechanism of GC to facilitate the screening of a population at high risk might be a feasible and efficient way to improve the current situation.

Many studies on the pathogenesis of GC have demonstrated that the development of GC is the result of combined mutations of multiple genes, like *TEAD4*‐*activated MNX1*‐*AS1* to promote the progression of GC cells via the *EZH2*/*BTG2* and the *miR*‐*6785*‐*5p*/*BCL2* axes.^6^, ^7^ LncRNAs do not participate in protein synthesis but have a direct biological function in the form of RNA.^8^, ^9^ Several studies have linked lncRNAs to the pathophysiological mechanism of various diseases, especially during tumorigenesis.^9^ It was recently discovered that RNAs and microRNAs (miRNAs) show mutual influence, ie, various types of RNAs such as lncRNA and miRNA "talk" to each other using microRNA response elements (MREs) to regulate gene expression, thus forming a competing endogenous RNA (ceRNA) network.^10^ As a ceRNA, lncRNA regulates gene expression at various levels by communicating with miRNAs to influence the initiation and progression of cancer and might be used as a biomarker for diagnosis and therapy.^11^

Through the lncRNA microarray analysis of GC tissues, we discovered that the expression of many lncRNAs is altered in GC, with the greatest changes found in *LINC00152* and *gastric cancer–associated transcript 3 (GACAT3)*.^12^, ^13^ *LINC00152* was reported to be associated with cell cycle progression and cell proliferation in various cancers. For example, it promotes the growth and invasion of cells in papillary thyroid carcinoma and regulates the *miR*‐*497*/*BDNF* axis^14^; it acts as a ceRNA by sponging miR‐193a/b‐3p to decrease *CCND1* expression and cell proliferation capacity in hepatocellular carcinoma^15^; finally, it promotes tumor growth through the *EGFR*‐mediated *PI3K*/*AKT* pathway in GC.^16^ However, the role of *LINC00152* in GC concerning other signaling pathways still needs further investigation. *HMGA1* is a protein closely related to tumor growth, infiltration, and metastasis and is highly expressed in various cancers.^17^ We have shown that lncRNA *GACAT3* promotes GC cell proliferation as a ceRNA of *HMGA1*, and its overexpression reduces apoptosis induced by the anticancer chemical compound cucurbitacin B.^12^ In this study, we investigated if *LINC00152* acts as a ceRNA to regulate *HMGA1*.

## 2 MATERIALS AND METHODS

### 2.1 Cell culture

Human gastric cancer cells AGS and BGC‐823 were cultured in RPMI 1640 medium (Corning, USA) containing 10% (v/v) fetal bovine serum (PAN, Germany) and 100 U/ml penicillin‐streptomycin (Sigma‐Aldrich, USA) at 37°C with 5% CO_2_.

### 2.2 miRNA transfection and siRNA knockdown

The mimics of miRNAs Let‐7a, miR‐103, miR‐196, miR‐128a, and miR‐138 as well as the negative control were synthesized by Shanghai GenePharma (Shanghai, China). The siRNA control, si*HMGA1*, and si*LINC00152* were prepared as described previously,^18^ and the miRNA mimics and siRNAs were transfected into the cells using Lipofectamine 2000 and RNAiMAX, respectively, according to the manufacturer's standard protocols (Invitrogen).

### 2.3 Quantitative reverse transcription‐polymerase chain reaction (qRT‐PCR)

RNA was extracted using TRIzol (Invitrogen), and the RNA concentration was assessed with Nanodrop 2000 (Thermo Scientific). A total of 500 ng RNA were reverse‐transcribed to cDNA with random primer using the GoScript Reverse Transcription System (Promega). For quantification, real‐time PCR analysis was performed using the LightCycler 480 SYBR Green I Master kit on a Light‐ Cycler 480 II (Roche). The relative fold changes were calculated by the comparative threshold cycle method, and β‐actin was used as the internal normalization control.

### 2.4 Cell proliferation assay

Cell proliferation was measured by cell number counting. Briefly, cells were seeded in 6‐well plates at 2 × 10^5^ cells/well and cultured. Cell numbers were counted using a hemocytometer every 24 h after transfection. Apoptotic or dead cells were excluded using 0.2% trypan blue (Sigma‐Aldrich) staining, and all experiments were performed 3 times.

### 2.5 Cell cycle analysis

The cell cycle staining (MultiSciences Biotech Co., Ltd.) was used to analyze the cell cycle by flow cytometry. In brief, siRNA‐treated cells were harvested and washed twice with ice‐cold phosphate‐buffered saline (PBS) and collected according to the previously described method.^19^ DNA content was analyzed on a BD FACS Calibular Flow Cytometer (BD Biosciences). Results were analyzed using the cell cycle analysis software ModFit LT (from Verity Software House, Inc.).

### 2.6 Western Blot

Cells were lysed using a standard RIPA lysis buffer supplemented with 2‐mM PMSF, and the extracted proteins were separated by sodium dodecyl sulfate polyacrylamide gel electrophoresis (12% SDS‐PAGE). Proteins were detected using antibodies against *HMGA1* (Active Motif Cat# 3961), *P27* (Affinity Biosciences Cat# AF6324), and β‐actin (Sigma‐Aldrich Cat# A1978).

### 2.7 Comprehensive analysis

The RNA‐Seq data and related clinical information from TCGA were downloaded in GDC API; there are 375 cancer samples and 32 normal samples. All tumor samples are from before treatment. The RNA expression of *HMGA1*, *LINC00152*, and *Let*‐*7a* was analyzed and compared with stomach adenocarcinoma (STAD) and evaluated the area under the curve (AUC). Using GSEA to analyze the KEGG pathways to investigate possible biological functions of *HMGA1*, the online database GEPIA (http://gepia.cancer‐pku.cn/index.html) was used to check the correlation analysis result between *LINC00152*/*HMGA1* by TCGA and GTEx database. The TIMER (https://cistrome.shinyapps.io/timer/)correlation module was used to perform correlation analysis between *HMGA1* and tumor‐infiltrating immune cells. The TISIDB (http://cis.hku.hk/TISIDB/index.php) was used to evaluate tumor and immune system interactions in 28 types of TILs across human cancers and the correlation between TILs and STAD. The Human Pathology Atlas project (HPA) (www.proteinatlas.org) provide immunohistochemistry on tissue containing 44 different tissue types. In our study, the HPA was used to analyze protein expression of *HMGA1* between normal and stomach cancer tissues.^20^

### 2.8 Statistical analysis

The SPSS software 18.0 was used for statistical analysis. Statistical significance and *p* values were determined by one‐way analysis of variance (ANOVA) and two‐sample independent *t* test, and related values are shown as mean ± SD.

## 3 RESULTS

### 3.1 *LINC00152* and *HMGA1* are suppressed by the same miRNAs

**FIGURE 1:**
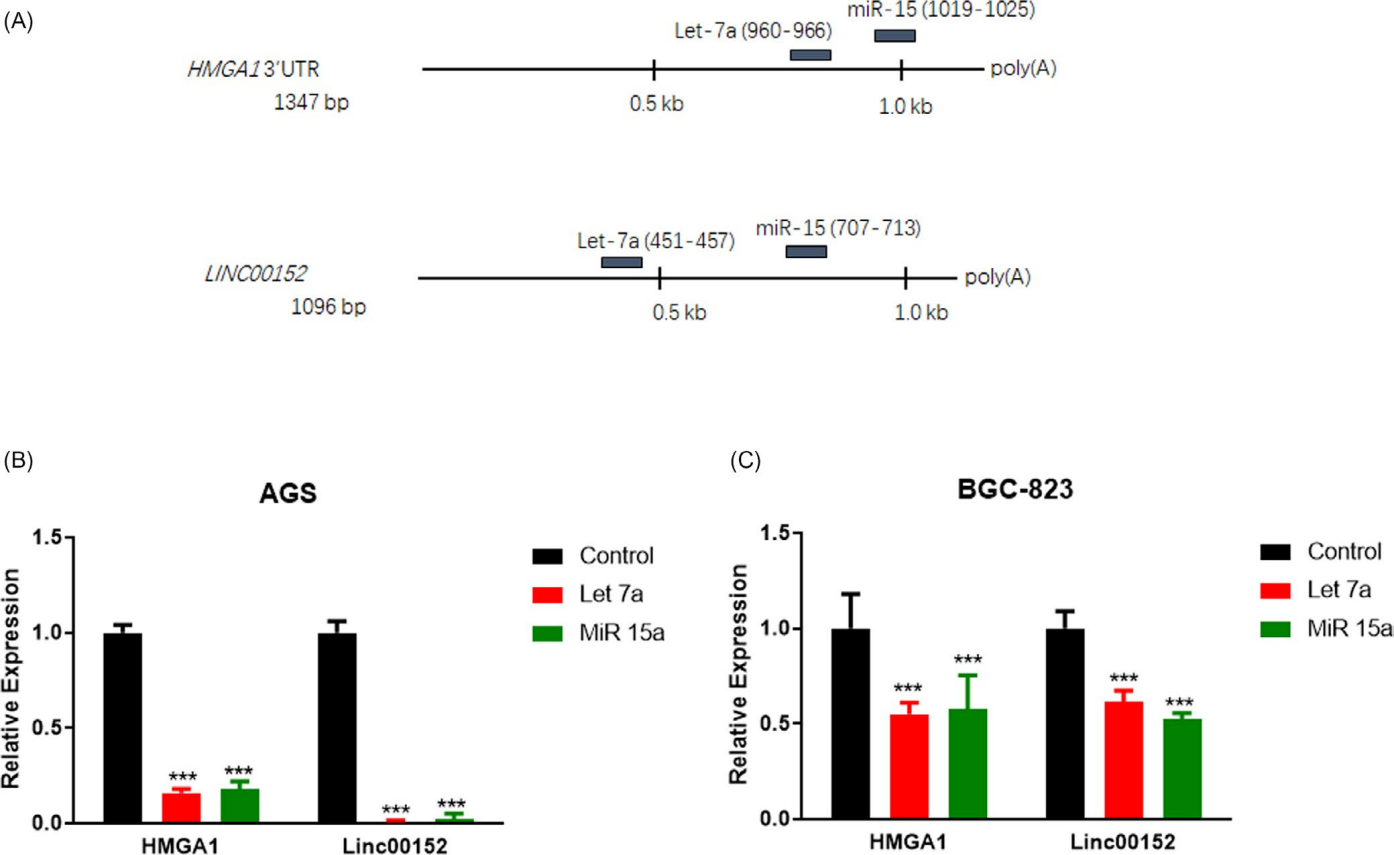
*LINC00152* and *HMGA1* are suppressed by the same miRNAs. (A) Common miRNAs that have putative binding sites in the *LINC00152* and *HMGA1* 3′‐UTR sequences are presented based on the analysis performed using microT, miRcode, and TargetScan. (B and C). Both *LINC00152* and *HMGA1* gene expression was inhibited by the common miRNAs. In vitro synthesized miRNA mimics of miR‐7a and miR‐15 were applied to human gastric cancer cells AGS and BGC‐823. *LINC00152* and *HMGA1* expression levels were determined by qRT‐PCR. Data represent the mean ± S.D. (*n* = 3). ***p < 0.001

The entire mRNA sequence of *LINC00152* and the 3′‐UTR sequence of *HMGA1* were obtained from the NCBI database (National Center for Biotechnology Information, https://www.ncbi.nlm.nih.gov/). We used the software microT (http://diana.imis.athena‐innovation.gr/DianaTools/ ), miRcode (http://www.mircode.org/mircode/ ), and TargetScan (http://www.targetscam.org/) to predict the binding sites of the miRNAs in *LINC00152* and the *HMGA1* 3'‐UTR. Two miRNAs, let‐7a and miR‐15, were found to have binding sites in both genes (Figure 1A). To verify the regulatory effects of the two miRNAs, mimics of let‐7a and miR‐15 were transfected into human GC cells AGS and BGC‐823 (Figure 1B, C). Both miRNA mimics decreased the expression of *LINC00152* and *HMGA1* in BGC‐823 cells two‐fold (Figure 1B), while a greater reduction in the expression of these two genes was observed in the AGS cells (Figure 1B), indicating that both genes are regulated by these two miRNAs.

### 3.2 *LINC00152* and *HMGA1* are reciprocally regulated

**FIGURE 2:**
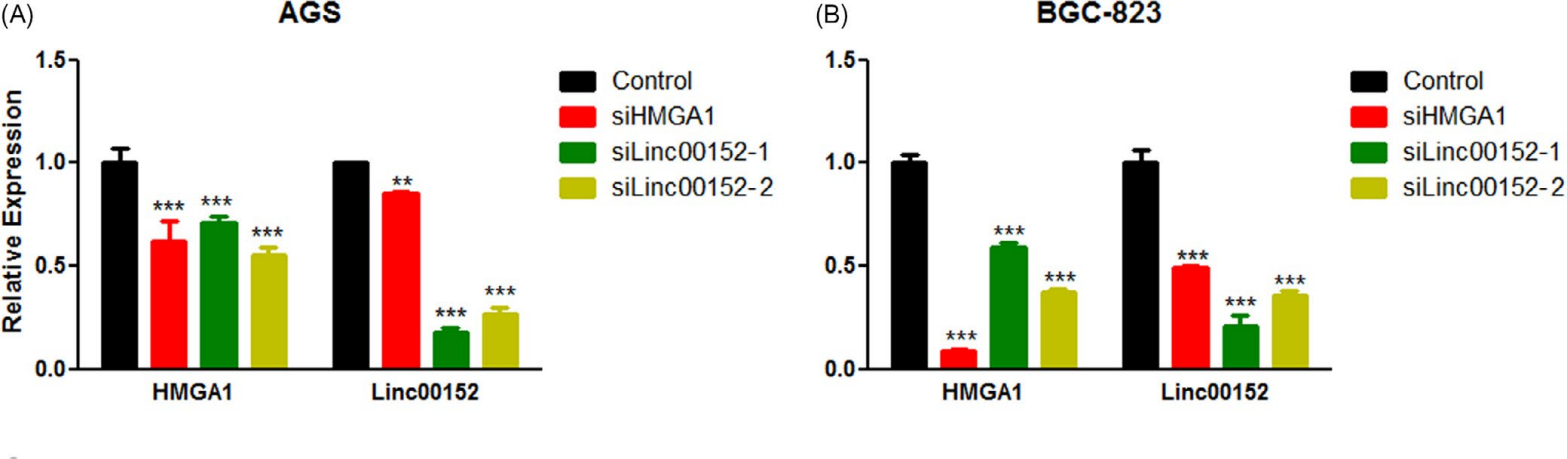
*HMGA1* and *LINC00152* are reciprocally regulated. (A and B). To further verify that *LINC00152* was used as the ceRNA of *HMGA1*, the relative expression of *LINC00152* and *HMGA1* in two GC cell lines (A) AGS, (B) BGC‐823) was measured by qRT‐PCR 48 h after siRNA transfection. Data represent the mean ± S.D. (*n* = 3). **p < 0.01; ***p < 0.001

The ceRNA hypothesis suggests that various types of RNA transcripts compete to bind to a common miRNA site, thereby affecting the level of free miRNAs and achieving mutual regulation.^10^ The results mentioned above showed that *LINC00152* and *HMGA1* share the same miRNA binding sites, and the inhibition of their expression by the same miRNAs is consistent with the ceRNA hypothesis. To further determine whether *LINC00152* is a ceRNA of *HMGA1*, we reduced the expression of *HMGA1* and *LINC00152* by specific siRNAs. The expression of *LINC00152* was significantly inhibited in *HMGA1* knockdown AGS (Figure 2A) and BGC‐824 (Figure 2B) cells. *HMGA1* expression decreased when *LINC00152* expression was inhibited (Figure 2). Thus, our results indicated that *LINC00152* acts as the ceRNA of *HMGA1*, and the two genes mutually regulate each other.

### 3.3 *HMGA1* and *LINC00152* promote cell proliferation in GC cells

**FIGURE 3:**
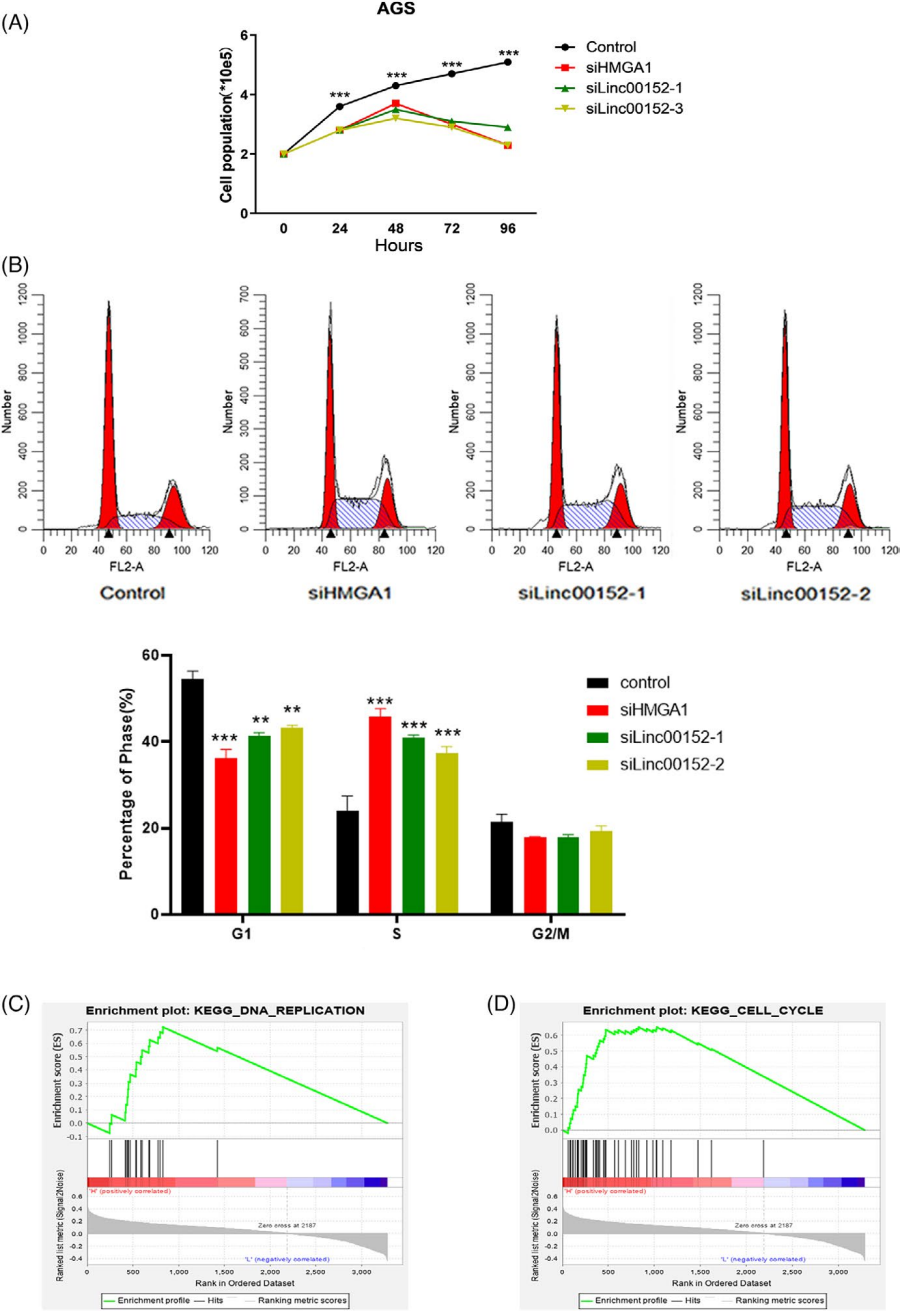
*HMGA1* and *LINC00152* promote cell proliferation in GC cells. (A) Cell growth was determined by cell number after knockdown of *LINC00152* and *HMGA1* in AGS cell. (B) Analysis of the effects of *LINC00152* and *HMGA1* knockdown on the cell cycle by flow cytometry 48 h after siRNA transfection in AGS cell. Data represent the mean ± S.D. (*n* = 3). (C and D) GSEA indicates that high expression of *HMGA1* is associated with the DNA replication (C) and cell cycle (D) in the TCGA database. **p < 0.01; ***p < 0.001

*HMGA1* has been reported to be highly expressed in GC and can promote tumor cell growth.^21^ We next tested whether *LINC00152* had similar functions as *HMGA1* in GC cells. Using specific siRNAs to reduce the expression of *HMGA1* and *LINC00152*, we found that cell proliferation was significantly inhibited in AGS cell (Figure 3A). Moreover, the proportion of cells arrested in the S phase nearly doubled in *HMGA1* knockdown (45.76%) and *LINC00152* knockdown (40.84% and 37.34%) cells, compared to the proportion of arrested cells in the control group (24.09%) (Figure 3B). Furthermore, GSEA in the TCGA database indicated that a high expression of HMGA1 was associated with DNA replication (*N* = 1.8, nominal *p*‐value = 0.001, FDR q‐value = 0.047) and cell cycle (*N* = 1.8, nominal *p*‐value = 0.012, FDR q‐value = 0.045) (Figure 3C, D). These results revealed that the downregulation of both *HMGA1* and *LINC00152* can inhibit cell proliferation by affecting cell cycle progression.

### 3.4 *HMGA1* and *LINC00152* regulate key cell cycle–related genes

**FIGURE 4:**
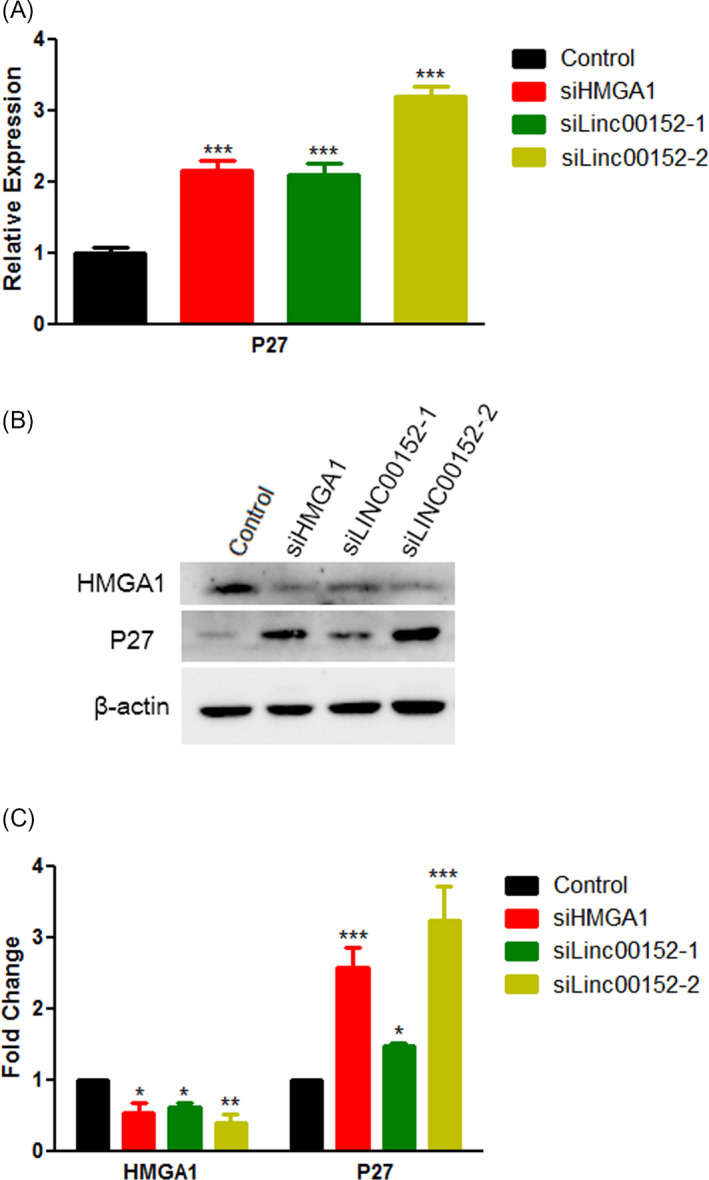
*HMGA1* and *LINC00152* regulate cell cycle progression. (A) The relative expression of *P27* was measured after siRNA transfection. (B) Western blot analysis of *HMGA1*, *P27*, and *β*‐*actin* 48 h after siRNA transfection. (C) The relative *HMGA1* and *P27* protein levels were quantified from B. Data represent the mean ± S.D. (*n* = 3). *p < 0.05; **p < 0.01; ***p < 0.001

To further elucidate the mechanism of how *LINC00152* and *HMGA1* block the proliferation of GC cells, we measured the expression levels of the cell cycle–related protein *P27* at both the RNA and protein levels. The mRNA expression of *P27* increased two‐fold in the *LINC00152* (Figure 4A) and *HMGA1* knockdown groups, and the *P27* protein level also increased (Figure 4B), compared to the levels in the control group. The protein expression of *HMGA1* decreased 1.5‐fold in the *LINC00152* siRNA‐treated cells, and the *P27* protein level increased consistently in the *HMGA1* siRNA‐treated cells (Figure 4C). These results further indicated the mutual regulation of these two genes and that they regulate the cell cycle by altering the expression of *P27*.

### 3.5 *HMGA1* and *LINC00152* are closely associated with GC

**FIGURE 5:**
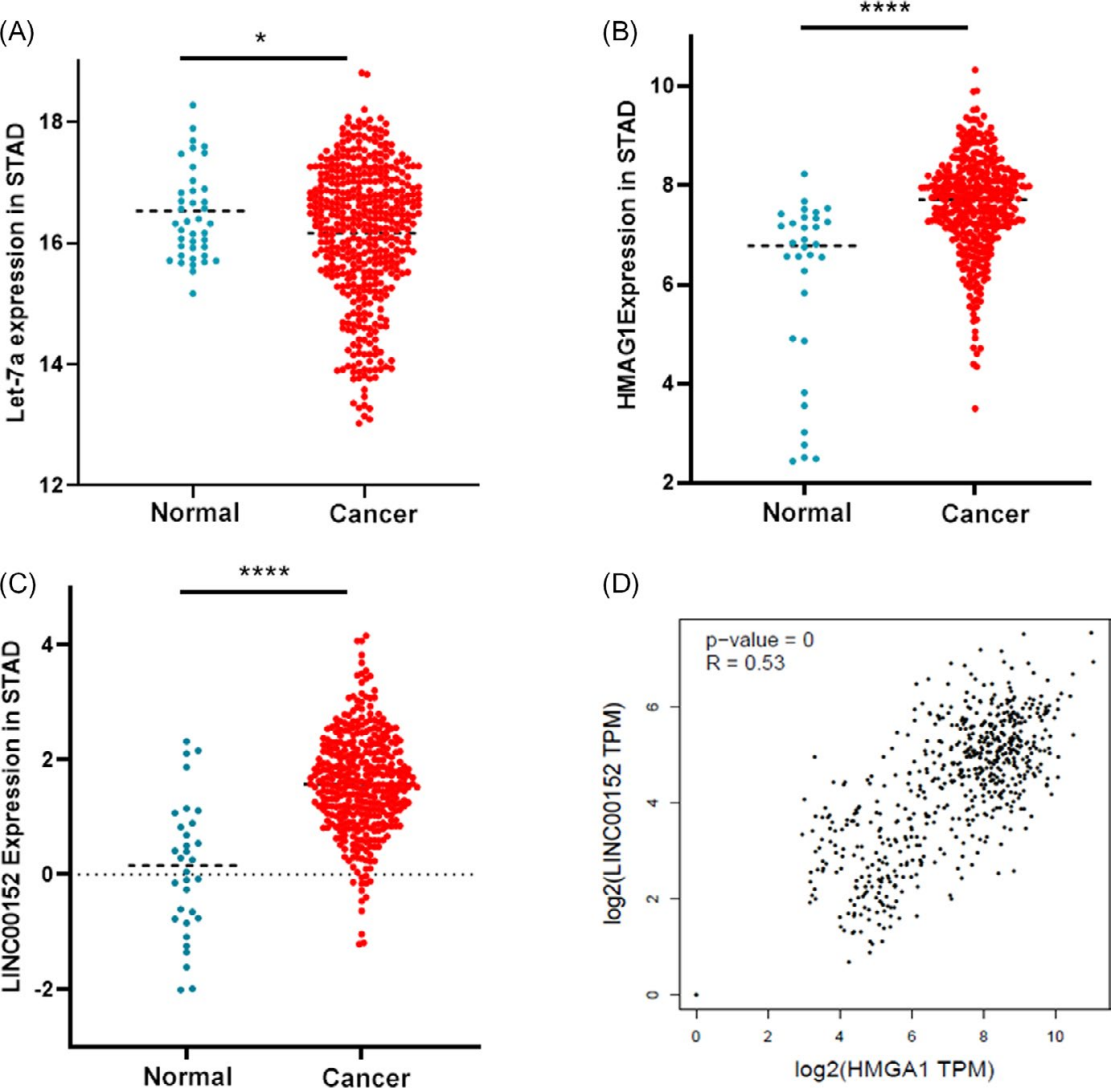
*HMGA1* and *LINC00152* positively correlated with STAD. (A–C). Comparison of *Let*‐*7a* (A), *HMGA1* (B) and *LINC00152* (C) RNA expression in STAD from TCGA database. (D) *HMGA1* expression is significantly positive correlations with *LINC00152* in stomach tumors from the GEPIA database. *p < 0.05; ****p < 0.0001

The TCGA database was used to investigate the expression of *HMGA1*, *LINC00152*, and *Let*‐*7a* in GC. As shown by scatter plots, a significantly decreased *Let*‐*7a* expression was observed in cancer tissues compared to that in normal tissues (*p* < 0.05), *HMGA1* and *LINC00152* were highly expressed in cancer tissues (Figure 5A–C). These clinical results from the database were consistent with the results of our cell experiments, suggesting that *Let*‐*7a*, *HMGA1*, and *LINC00152* affect GC by forming a ceRNA network. Additionally, a positive correlation between *HMGA1* and *LINC00152* in stomach tumors was detected from the GEPIA database (Figure 5D).

**FIGURE 6:**
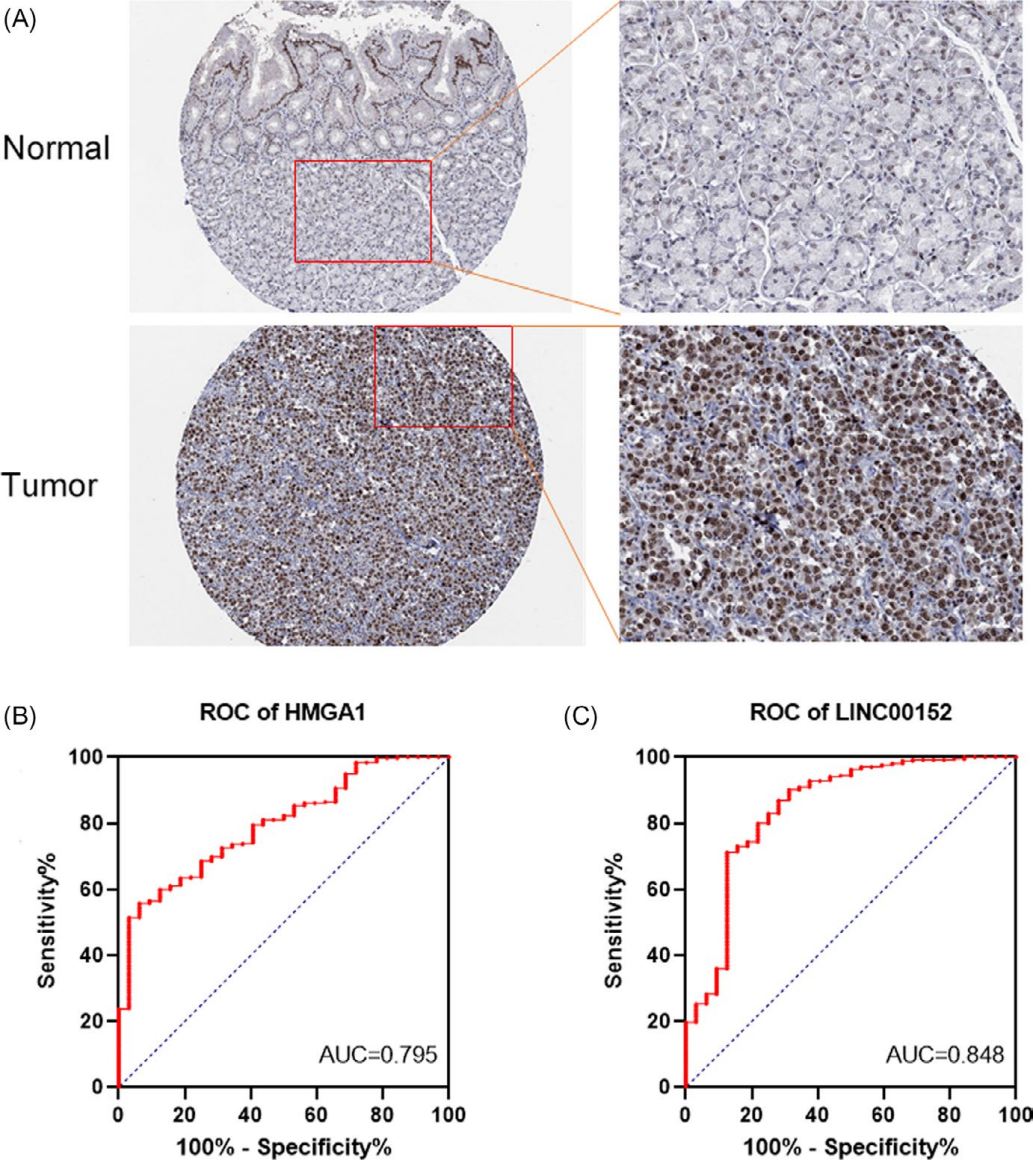
*HMGA1* and *LINC00152* could be diagnostic markers. (A) *HMGA1* expression in the stomach by immunohistochemical staining in HPA. (B and C) ROC curves of *HMGA1* (B) *LINC00152* (C) in STAD from TCGA database

Immunohistochemistry (IHC) staining indicated that *HMGA1* protein expression was higher in tumor tissues than in nontumor tissues from the HPA (Figure 6A). After integrating the clinicopathological parameters and gene expression profiles to analyze the potential ability of *HMGA1* and *LINC00152*, the AUC indices for the *HMGA1* and *LINC00152* were found to be 0.795 and 0.848 (Figure 6B, C). These results suggested that *HMGA1* and *LINC00152* might be used as diagnostic markers of STAD.

### 3.6 *HMGA1* influences immune infiltration and is associated with tumor‐infiltrating lymphocytes

**FIGURE 7:**
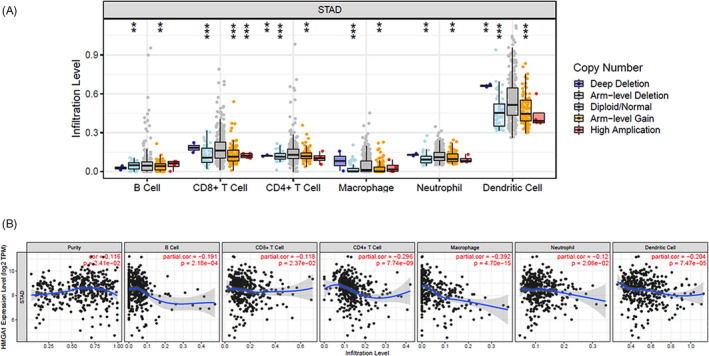
*HMGA1* impact immune infiltration. (A) CNA of *HMGA1* was significantly correlated with immune infiltration levels of several immune cells in STAD from TIMER database. (B) The negative correlation between *HMGA1* expression and immune infiltration levels of several immune cells. **p < 0.01; ***p < 0.001

Tumor progression is associated with the suppression of antitumor immunity.^22^ As *HMGA1* can promote cell proliferation during cancer development, we used the TIMER database to analyze the impact of *HMGA1* in immune infiltration, after determining the multiple types of copy number alterations (CNAs) of *HMGA1* that were significantly correlated with the infiltration levels of several immune cells in STAD, both in *HMGA1* deletion and amplification (Figure 7A and Table S1). We next performed correlation analysis between *HMGA1* and immune infiltration level for STAD. *HMGA1* expression showed a significant negative correlation with the levels of B cells, CD8 + T cells, CD4 + T cells, macrophages, neutrophils, and dendritic cells (*p *< 0.001, Figure 7B), leading to a general decrease in immune infiltration.

**FIGURE 8:**
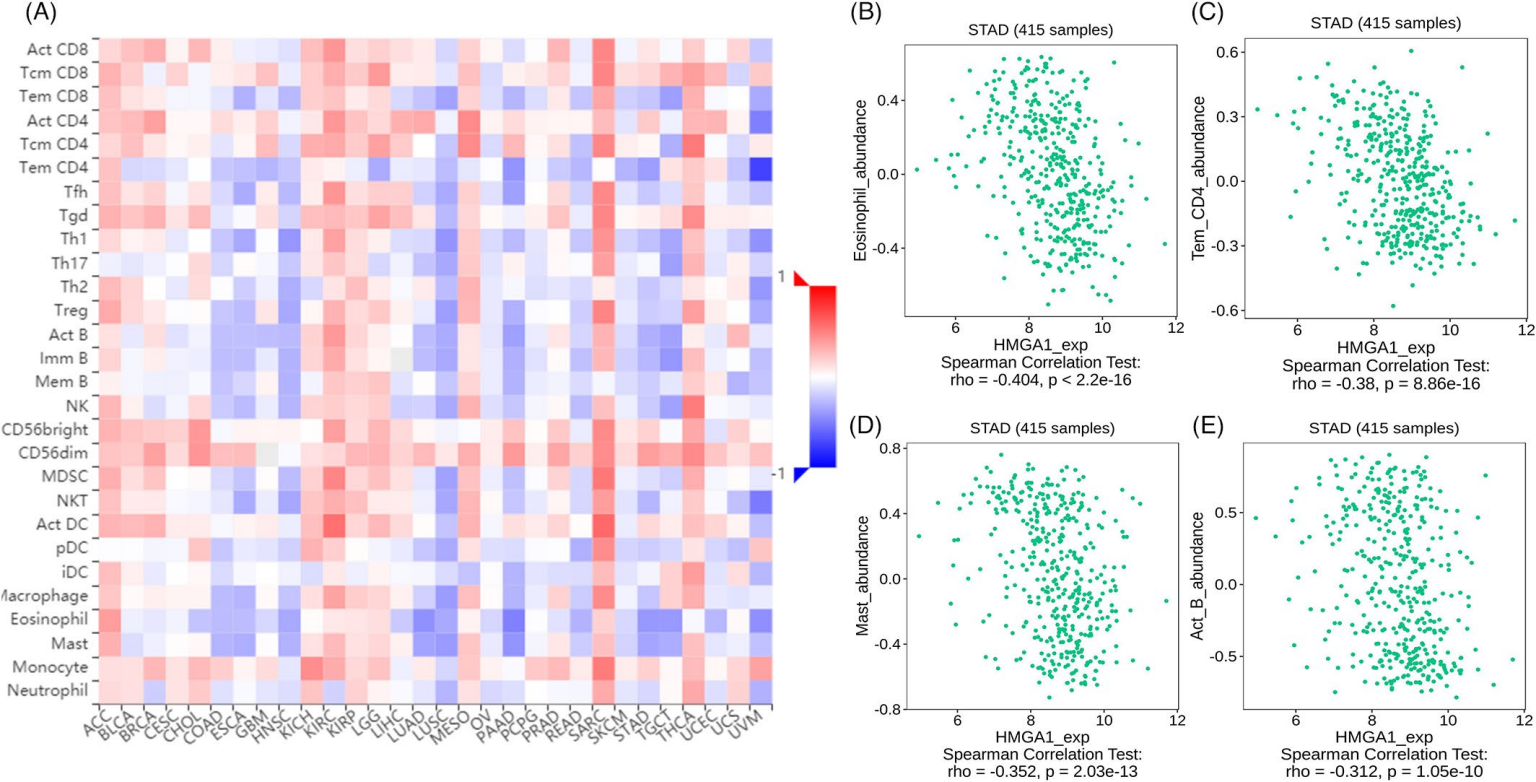
The negative correlation between *HMGA1* and TILs. (A) Relations between expression of *HMGA1* and 28 types of TILs across human cancers from TISIDB database. *HMGA1* significantly negative correlated with the abundance of eosinophil (B), Tem_CD4 (C), Mast (D), and Act_B (E)

Additionally, we further assessed the possible correlations between 28 types of tumor‐infiltrating lymphocytes (TILs) through the TISIDB database. The results showed significant correlations between *HMGA1* and the 28 types of TILs found in human cancers (Figure 8A). *HMGA1* showed significantly negative correlations with the abundance of eosinophil (rho = –0.404, *p* < 0.001), Tem CD4 (rho = 0.485, *p* < 0.001), Mast (rho = −0.352, *p* < 0.001), and Act B (rho = −0.312, *p* < 0.001) (Figure 8B–E). These results indicated that *HMGA1* has the potential to regulate immune infiltration in GC.

## 4 DISCUSSION

LncRNA was considered a junk gene until the ceRNA hypothesis was proposed, which suggests that lncRNA, cirRNA, and miRNA can interact with other mRNAs through the ceRNA mechanism to regulate cellular functions.^10^, ^23^ Moreover, the dysregulation of lncRNAs may disrupt cellular functions and lead to diseases, including cancer.^23^ For example, lnc‐ATB was found to promote tumor progression by interacting with *miR*‐*141*‐*3p*/*TGF*‐*β2* in a ceRNA‐mediated manner, and high expression of *LNC*‐*ATB* indicates a poor prognosis in GC patients.^24^ In this study, we found that *HMGA1* shares common miRNA binding sites (*let*‐*7a* and *miR*‐*15*) with *LINC00152*, and that the *HMGA1* and *LINC00152* expression are mutually regulated.

We used the TCGA data to verify our hypothesis that the expression of miRNA *Let*‐*7a* in gastric cancer tissue was downregulated, and the expression of *HMGA1* and *LINC00152* was upregulated, which showed that our previous hypothesis, i.e., *HMGA1*‐*Let*‐*7a*‐*LINC00152* is a ceRNA, was correct. Moreover, the expression of RNA and protein indicated the value of *HMGA1* and *LINC00152* as diagnostic markers.

*HMGA1* is a transcriptional regulatory molecule involved in tumorigenesis, affecting the normal growth and differentiation of cells, gene transcription and regulation, cell cycle progression, and DNA replication and repair in various ways.^25^ Normally, the expression level of *HMGA1* is very low, but *HMGA1* is a cancer‐promoting gene that is overexpressed in many malignant tumors.^26^ Previous studies have found that *HMGA1* is overexpressed in GC cells and promotes their proliferation.^27^ In this study, we also found that *HMGA1* promoted GC cell proliferation and that the cell cycle was arrested in the S phase when *HMGA1* expression was inhibited. The main function of *P27* is to control the cell cycle by inhibiting the progression of cells from the G1 phase to the S phase after binding to *cyclin*/*Cdk2*.^28^ Thus, we selected *P27* to elucidate the role of *HMGA1* and *LINC00152* in cell cycle and proliferation. As a ceRNA of *HMGA1*, *LINC00152* has a similar function in GC cell proliferation. Furthermore, we found that the cell cycle inhibitor *P27* was upregulated in AGS cells after the downregulation of *HMGA1*/*LINC00152*, thereby blocking cell growth. The results indicated that low levels of *HMGA1* and *LINC00152* arrest cell cycle progression and inhibit GC cell proliferation, suggesting that *LINC00152* promotes tumor growth and can form a ceRNA network with *HMGA1* to regulate the proliferation of GC cells.

In the tumor microenvironment, immune cells are crucial elements and significantly influence tumor progression.^29^ Our study used the TIMER and TISIDB databases to reveal the connections between the expression of *HMGA1* and immune infiltration levels in STAD. We found that *HMGA1* has a strong negative correlation with macrophages and CD4 + T cells. Macrophages can perform phagocytosis of immune cells. Changes in the levels of immune infiltration indicated that the ability of macrophages was affected by the expression of HMGA1. Furthermore, we found that the expression of *HMGA1* also had a very strong negative correlation with the abundance of TILs, particularly eosinophil, Tem_CD4, Mast, and Act_B cells. Thus, combining the results of the biosynthesis analysis and the cell experiments, we hypothesized that *HMGA1* might promote the proliferation of GC cells, possibly by reducing the infiltration level of immune cells and the content of TILs. Additionally, we found that *LINC00152* has the same function as *HMGA1* in GC cells, and *LINC00152* might also promote GC proliferation by affecting immune cells.

## 5 CONCLUSION

In summary, our findings provided mechanistic insights into the role of *LINC00152* as a ceRNA to regulate *HMGA1* expression in GC cells. *LINC00152* thus promotes the proliferation of GC cells by regulating the expression of the cell cycle inhibitor *P27*. The immune‐related bioinformatics results showed that *HMGA1* and *LINC00152* play a role in the cell cycle and proliferation of GC cells, perhaps by reducing the infiltration of immune cells and the content of TILs. Moreover, the bioinformatics results indicated that *HMGA1* and *LINC00152* might be used as diagnostic markers of GC.

## Supporting information

Table S1

## Data Availability

All data included in this study are available upon request by contact with the corresponding author.
