# Supplementary material for: LINC00152 acts as a competing endogenous RNA of HMGA1 to promote the growth of gastric cancer cells
Source: J Clin Lab Anal. 2022 Jan 11;36(2):e24192. doi: 10.1002/jcla.24192 (PMC8841176; doi:10.1002/jcla.24192)
Supplement: Supplementary file 1 — Table S1 [file JCLA-36-e24192-s001.docx]

**Supplementary Table 1. Correlations between immune infiltration levels of different immune cells and copy number alteration (CNA) of HMGA1.**

| Cancer | Immune Cells | CNA_level | P value |
| --- | --- | --- | --- |
| STAD | B Cell | Deep Deletion | 0.191612243 |
| STAD | B Cell | Arm-level Deletion | 0.002799639 |
| STAD | B Cell | Diploid/Normal | 1 |
| STAD | B Cell | Arm-level Gain | 0.001211871 |
| STAD | B Cell | High Amplication | 0.481225047 |
| STAD | CD8+ T Cell | Deep Deletion | 0.911675948 |
| STAD | CD8+ T Cell | Arm-level Deletion | 1.81E-05 |
| STAD | CD8+ T Cell | Diploid/Normal | 1 |
| STAD | CD8+ T Cell | Arm-level Gain | 1.51E-05 |
| STAD | CD8+ T Cell | High Amplication | 0.000869997 |
| STAD | CD4+ T Cell | Deep Deletion | 0.003060018 |
| STAD | CD4+ T Cell | Arm-level Deletion | 2.83E-05 |
| STAD | CD4+ T Cell | Diploid/Normal | 1 |
| STAD | CD4+ T Cell | Arm-level Gain | 0.002442467 |
| STAD | CD4+ T Cell | High Amplication | 0.102372131 |
| STAD | Macrophage | Deep Deletion | 0.764259538 |
| STAD | Macrophage | Arm-level Deletion | 0.000740784 |
| STAD | Macrophage | Diploid/Normal | 1 |
| STAD | Macrophage | Arm-level Gain | 0.001276704 |
| STAD | Macrophage | High Amplication | 0.397407836 |
| STAD | Neutrophil | Deep Deletion | 0.695505706 |
| STAD | Neutrophil | Arm-level Deletion | 0.001572638 |
| STAD | Neutrophil | Diploid/Normal | 1 |
| STAD | Neutrophil | Arm-level Gain | 0.00423558 |
| STAD | Neutrophil | High Amplication | 0.107151858 |
| STAD | Dendritic Cell | Deep Deletion | 0.004944743 |
| STAD | Dendritic Cell | Arm-level Deletion | 0.000138595 |
| STAD | Dendritic Cell | Diploid/Normal | 1 |
| STAD | Dendritic Cell | Arm-level Gain | 9.60E-06 |
| STAD | Dendritic Cell | High Amplication | 0.125907278 |
